# Diagnostic performance of new BAST score versus FIB-4 index in predicating of the liver fibrosis in patients with metabolic dysfunction-associated steatotic liver disease

**DOI:** 10.1186/s40001-024-02032-x

**Published:** 2024-09-14

**Authors:** Eman Helal, Fatma Elgebaly, Nasser Mousa, Sherif Elbaz, Mostafa Abdelsalam, Eman Abdelkader, Amr El-Sehrawy, Niveen El-wakeel, Ola El-Emam, Manal Hashem, Alaa Elmetwalli, Shimaa Mansour

**Affiliations:** 1 Department of Tropical Medicine, Faculty of Medicine, Tanta University, Tanta, Egypt; 2 Tropical Medicine Department, Mansoura University, Mansoura, Egypt; 3 Internal Medicine Department, Jacobs School of Medicine and Biomedical Sciences, Buffalo University, New York, USA; 4 Internal Medicine Department, Mansoura University, Mansoura, Egypt; 5 Alameen General Hospital, Taif, Kingdom of Saudi Arabia; 6 Medical Microbiology and Immunology Department, Mansoura National University, Mansoura, Egypt; 7 Medical Microbiology and Immunology Department, Faculty of Medicine, Delta University for Science and Technology, Mansoura, Egypt; 8 Clinical Pathology Department, Mansoura University, Mansoura, Egypt; 9 Internal Medicine Department, Zagazig University, Zagazig, Egypt; 10 Department of Clinical Trial Research Unit and Drug Discovery, Egyptian Liver Research Institute and Hospital (ELRIAH), Mansoura, Egypt; 11 Microbiology Division, Higher Technological Institute of Applied Health Sciences, Egyptian Liver Research Institute and Hospital (ELRIAH), Mansoura, Egypt

**Keywords:** BAST score, MASLD, Non-invasive diagnosis, FIB4 score, Transient elastography

## Abstract

**Background and aim:**

Metabolic dysfunction-associated steatotic liver disease (MASLD) formerly known as non-alcoholic fatty liver disease (NAFLD) is the most common liver condition globally. The FIB-4 test is used to detect fibrosis in fatty liver disease but has limited accuracy in predicting liver stiffness, resulting in high rates of false positives and negatives. The new BAST scoring system, incorporating waist circumference, AST, and BMI, has been developed to assess the presence of fibrosis in NAFLD patients. This study compares the effectiveness of BAST and FIB-4 in predicting liver fibrosis in MASLD patients.

**Patients and methods:**

The study included 140 non-diabetic MASLD patients who underwent transient elastography measurement. BAST score and FIB-4 were calculated for each patient. Patients were grouped based on fibrosis severity; F1, F2, and F3–F4. The sensitivity and specificity of the BAST score and FIB-4 were assessed using receiver operating characteristic curves.

**Results:**

The BAST score increased significantly with fibrosis progression from F1 to F3–F4. In differentiating advanced fibrosis (F2–F3) from mild/moderate fibrosis (F1–F2), the BAST score at cutoff ≤ − 0.451 showed better diagnostic performance with 90.70% sensitivity, 74.07% specificity, 84.8% PPV and 83.3% NPV compared to FIB-4 that had 60.47% sensitivity, 50.0% specificity, 65.8% PPV and 44.3% NPV. Similarly, for differentiating between F1 and F2 fibrosis, the BAST score at cutoff ≤ − 1.11 outperformed FIB-4, with 80.23% sensitivity, 79.49% specificity, 89.6% PPV and 64.6% NPV, while FIB-4 had 59.30% sensitivity, 51.28% specificity, 72.9% PPV and 36% NPV.

**Conclusions:**

The BAST score is a better predictor of liver fibrosis in MASLD compared to FIB-4, especially in cases of advanced fibrosis or cirrhosis.

## Introduction

The classification of non-alcoholic fatty liver disease (NAFLD) has been debated in the medical community. Originally called NAFLD, it was later renamed metabolic dysfunction-associated fatty liver disease (MAFLD) and, most recently, metabolic dysfunction-associated steatotic liver disease (MASLD) to emphasize the metabolic component of the condition. These terms are defined by diagnostic criteria related to metabolic risk factors. However, the distinctions in characteristics and mortality rates between NAFLD, MAFLD, and MASLD are still unclear [1]. MASLD has a new nomenclature according to a recent multi-society Delphi meeting [2]; however, more than 99.5% of patients with NAFLD met the MASLD criteria. Since there have been significant changes in lifestyle throughout the past 20 years, MASLD has emerged as the most common liver ailment [3, 4]. Liver biopsy is a gold method for diagnosing liver fibrosis, but it is invasive and has limitations such as cost and potential complications. Non-invasive methods, including scoring systems and imaging techniques, are now available to assess liver fibrosis [5]. The FIB-4 score, which combines age, aspartate aminotransferase (AST) levels, platelet count, and alanine aminotransferase (ALT) levels, is a useful blood-based biomarker for evaluating and risk stratification of fibrosis without the need for a biopsy in patients with fatty liver disease. However, the FIB-4 index may not be effective in accurately detecting advanced liver fibrosis in individuals with diabetes and fatty liver disease [6, 7]. The FIB-4 index may not reliably detect individuals with liver stiffness measurements of 8 kPa or higher, potentially leading to false negative results in about 20% of cases [8]. A new scoring system called the BAST score, which considers waist circumference, AST levels, and BMI, has been developed to assess the presence of fibrosis in NAFLD patients. The BAST score was derived by integrating three parameters: waist circumference, body mass index (BMI), and AST levels. The score was initially validated internally in patients with diabetic NAFLD and has shown promising result in predicting liver disease (LSM > 8.1 kPa) due to NAFLD better than FIB-4 and NAFLD fibrosis score in diabetic patients. Likewise, the BAST score performed better when predicting the presence of advanced fibrosis (LSM > 12 kPa) in the same population [9]. These parameters were selected based on their established association with the severity of liver disease. Waist circumference and BMI are well-known risk factors for MASLD and are commonly used indicators for detecting obesity or abdominal obesity [2]. AST levels are markers of hepatocellular injury, and their increase is associated with advancing hepatic fibrosis and increased with high body mass index [10].

This study aims to evaluate the diagnostic value of the BAST score versus the FIB-4 index in the prediction of liver fibrosis in patients with MASLD.

## Patients and methods

This cross-sectional study involved 140 non-diabetic patients with MASLD who were over 18 years. To diagnose MASLD, liver steatosis must be present along with at least one of the following cardiometabolic criteria; impaired glucose regulation, type 2 diabetes, overweight or obesity, hypertension, or dyslipidemia [2].

The patients were seen at the outpatient Departments of Tanta and Mansoura Tropical Medicine, as well as the outpatient departments of Mansoura and Zagazig Internal Medicinefrom October 2023 to Jun 2024. The study was conducted after obtaining institutional ethical approval (approval code: 3624PR321/9/23) and following the ethical guidelines of the 1975 Declaration of Helsinki.

*Exclusion criteria* Patients under the age of eighteen, those with a history of diabetes mellitus (based on medical history), and individuals with a history of alcohol consumption exceeding 20 g for women and 30 g for men were excluded. The exclusion of diabetic patients was aimed at reducing confounding factors, as diabetes can independently affect liver fibrosis and related metabolic parameters. However, it is important to note that HbA1c was not measured in this study, raising the possibility of undiagnosed diabetes within the cohort. All patients who met the inclusion criteria underwent a comprehensive assessment, including measurements of height, weight, BMI, and waist circumference. Waist circumference was measured using a standardized protocol recommended by the World Health Organization (WHO). The measurement was taken at the midpoint between the lower margin of the last palpable rib and the top of the iliac crest, with the patient standing and breathing normally. This method ensures accuracy and consistency in assessing central obesity. Clinical examinations were conducted, including measurements of height, weight, BMI, and waist circumference. Basic investigations, such as complete blood count (CBC), total bilirubin, albumin, liver enzymes (AST and ALT), kidney function tests, and lipid profile tests, were performed. CBC was conducted using an automated hematology analyzer (Sysmex Corporation, Japan). AST and ALT measurements were based on the IFCC reference methods with pyridoxal-5-phosphate (P5P) to ensure accuracy. The biochemical tests were performed using a fully automated chemistry analyzer (Roche Diagnostics, Basel, Switzerland).

*Ultrasound imaging* Conventional B-mode ultrasonography is commonly used in population studies to diagnose fatty liver. Diagnosis is based on ultrasound parameters such as parenchymal brightness, liver-to-kidney contrast, deep beam attenuation, bright vessel walls, and gallbladder wall definition. Qualitative grades range from mild to severe, graded from 0 to 3. Grade 1 (mild) shows a slight increase in fine echoes in the liver parenchyma, grade 2 (moderate) shows a moderate increase with slightly impaired visualization of vessels, and grade 3 (marked) shows a significant increase with poor visualization of vessels and the diaphragm [10].

The scores were calculated using the following equations:

The FIB-4 index = Age (years) × AST (U/L)/ [PLT (10^9^/L) × ALT^1/2^ (U/L)] [12].

The BAST score = 0.086 × (Waist circumference/ cm) + 0. 08 × (BMI/ kg/m^2^) + 0.025 × (AST/ IU/L) − 14. 607 [9].

### FibroScan; transient elastography (TE)

At the index time of the study, every patient was subjected to anthropometric measurements and laboratory investigation. Transient Elastography was conducted using FibroScan 502 (Echosens) and three portable Echosens Mini Systems. The FibroScan^®^ probe consists of a 3.5 MHz ultrasonic transducer mounted on a low-amplitude vibrator with a frequency of 50 Hz and an amplitude of 2 mm peak-to-peak. Liver stiffness measurement (LSM) and Controlled Attenuation Parameter (CAP) were performed by a blinded experienced operator. Only results with ten accurate measurements, an interquartile range (IQR)/median liver stiffness ratio of less than 30%, and a success rate exceeding 60% were considered reliable. Both LSM and CAP readings were taken from the same liver parenchyma region (depth between 25 and 65 mm) and reported in Kpa and dB/m, respectively [13]. According to studies, fibrosis stages are classified as follows: F0 (1–6 kPa), F1 (6.1–7 kPa), F2 (7–9 kPa), F3 (9.1–10.3 kPa), and F4 (≥ 10.4 kPa) [14, 15]. The cutoff values for liver steatosis (S) based on Controlled Attenuation Parameter (CAP) are as follows: SO for no steatosis (≤ 237 dB/m), S1 for mild steatosis (237.0 to 259.0 dB/m), S2 for moderate steatosis (259.0 to 291.0 dB/m), and S3 for severe steatosis (291.0 to 400.0 dB/m) [16]. In cases of high BMI (≥ 30 kg/m^2^), the examination was conducted using the XL probe by two experienced operators.

### Statistical analysis of the data

The data were fed into the computer and analyzed with the IBM SPSS software package version 20.0. IBM Corporation, Armonk, New York. Numbers and percentages were used to represent categorical data. The Chi-square test was used to compare the two Groups. Alternatively, the Kolmogorov–Smirnov test was used to determine normality for continuous data. Range (minimum and maximum), mean, standard deviation, and median were used to express quantitative data. The one-way ANOVA test was used to compare the various studied Groups, followed by the Post Hoc test (Tukey) for pairwise comparison. For non-normally distributed quantitative variables, the Kruskal–Wallis test was used, followed by the Post Hoc test (Dunn’s for multiple comparisons test) for pairwise comparison. The Spearman coefficient is used to calculate the correlation between two abnormally distributed quantitative variables. The significance of the obtained results was determined at a 5% level.

## Results

The present study included 140 non-diabetic MASLD patients. They were divided according to the degree of fibrosis based on FibroScan into three Groups: Group I (F1) included 86 patients (61.4%) with mild fibrosis, Group II (F2) included 39 patients (27.9%) with moderate fibrosis, and Group III (F3, F4) included 15 patients with advanced fibrosis/cirrhosis (10.7%). The mean age of the patients was 48.35 ± 7.9 years, with no significant difference in age between the three Groups. Seventy-five patients were females (53.57%), while 65 patients were males (46.43%), with no statistically significant difference between the studied Groups.

Table 1 shows a statistically significant increase in body mass index and waist circumference in Group II and Group III compared to Group I. There was no significant change when comparing Group II to Group I. In comparing the laboratory findings of the studied Groups, patients in Group III had a statistically significant increase in ALT and AST compared to the other two Groups (*p* value < 0.001). In addition, Group III had a significantly lower platelet count compared to Group I and Group II. Serum albumin, bilirubin, triglycerides, and high-density lipoprotein levels did not show significant differences between all Groups. The BAST score was significantly higher in Group III than in Group I and Group II and also significantly higher in Group II than in Group I. The FIB-4 score was significantly higher in Group III compared to Group I, with no significant changes between Group I and Group II and Group II and Group III. Regarding steatosis measured by CAP, there was a significant increase in Group III compared to Group I and Group II, with no significant changes between Group I and Group II.

**Table 1:** Demographic and Laboratory data of patients according to the stage of fibrosis by FibroScan

	Group I (F1) (N = 86)	Group II (F2) (N = 39)	Group III (F3–F4) (N = 15)	*p* value
Sex				
Male	41 (47.70%)	17 (43.60%)	7 (46.70%)	0.914
Female	45 (52.30%)	22 (56.40%)	8 (53.30%)	
Age/y (M ± SD)	48 ± 8.29	47.62 ± 6.60	52.27 ± 8.17	0.123
Body mass index (Kg/m^2^) M ± SD	33.75 ± 5.93	38.82 ± 8.20	40.31 ± 8.20	P1 < 0.001 P2 = 0.003 P3 = 0.641
Waist circumference (cm) M ± SD	105.90 ± 11.07	119.90 ± 9.25	125.20 ± 6.92	P1 < 0.001 P2 < 0.001 P3 = 0.207
ALT (U/L) Mean ± SD	30.44 ± 14.05	35.54 ± 19.62	71 ± 38.28	P1 = 0.278 P2 < 0.001 P3 < 0.001
AST (U/L) Mean ± SD	29.28 ± 12.76	36.34 ± 18.19	66.53 ± 30.91	P1 = 0.051 P2 < 0.001 P3 = 0.003
Albumin (g/dL) Median (Min.–Max.)	4 (3.50 – 5.90)	4.10 (3.20 – 5.80)	4.00 (3.20 – 5)	P1 = 0.491 P2 = 0.252 P3 = 0.471
Total bilirubin (mg/dL) Mean ± SD	0.74 ± 0.22	0.79 ± 0.14	0.73 ± 0.24	P1 = 0.459 P2 = 0.973 P3 = 0.601
PLT (× 10^3^/μL) Mean ± SD	224.70 ± 44.22	225.20 ± 27.09	186 ± 50.43	P1 = 0.997 P2 = 0.003 P3 = 0.006
Triglyceride (mg/dL) Mean ± SD	179.20 ± 41.52	189.80 ± 36.79	196.50 ± 63.62	P1 = 0.412 P2 = 0.723 P3 = 0.862
HDL (mg/dL): Mean ± SD	44.86 ± 7.80	44.33 ± 7.42	40.10 ± 8.11	P1 = 0.934 P2 = 0.075 P3 = 0.172
BAST: Mean ± SD	− 2.07 ± 1.27	− 0.28 ± 0.97	1.05 ± 0.71	P1 < 0.001 P2 < 0.001 P3 = 0.015
FIB4: M ± SD	1.18 ± 0.45	1.26 ± 0.38	2.30 ± 1.31	P1 = 0.234 P2 = 0.003 P3 = 0.053
S1	14 (16.30%)	1(2.60%)	1 (6.70%)	P1 = 0.086
S2	27 (31.40%)	13 (33.30%)	0 (00%)	P2 = 0.006
S3	45 (52.30%)	25 (64.10%)	14 (93.30%)	P3 = 0.015

*ALT* alanine aminotransferase, *AST* aspartate aminotransferase, *PLT* platelet, *WBCs* white blood cell, *HDL* high density lipoprotein, *S* grade steatosis between Group 2 and Group 3

P1: *p* value for comparing between Group 1 and Group 2

p2: *p* value for comparing between Group 1 and Group 3

p3: *p* value for comparing SD: Standard deviation

Table 2 shows that there was a significant positive correlation between the BAST score and degree of fibrosis (< 0.001). However, no correlation between BAST and FIB-4 was found.

**Table 2:** Correlation between BAST sore and FibroScan and FIB-4 index

	Bast	
	r_s_	*p*
FibroScan	0.644	< 0.001
FIB4	0.147	0.140

r_s_: Spearman coefficient, n: number

**Fig. 1:**
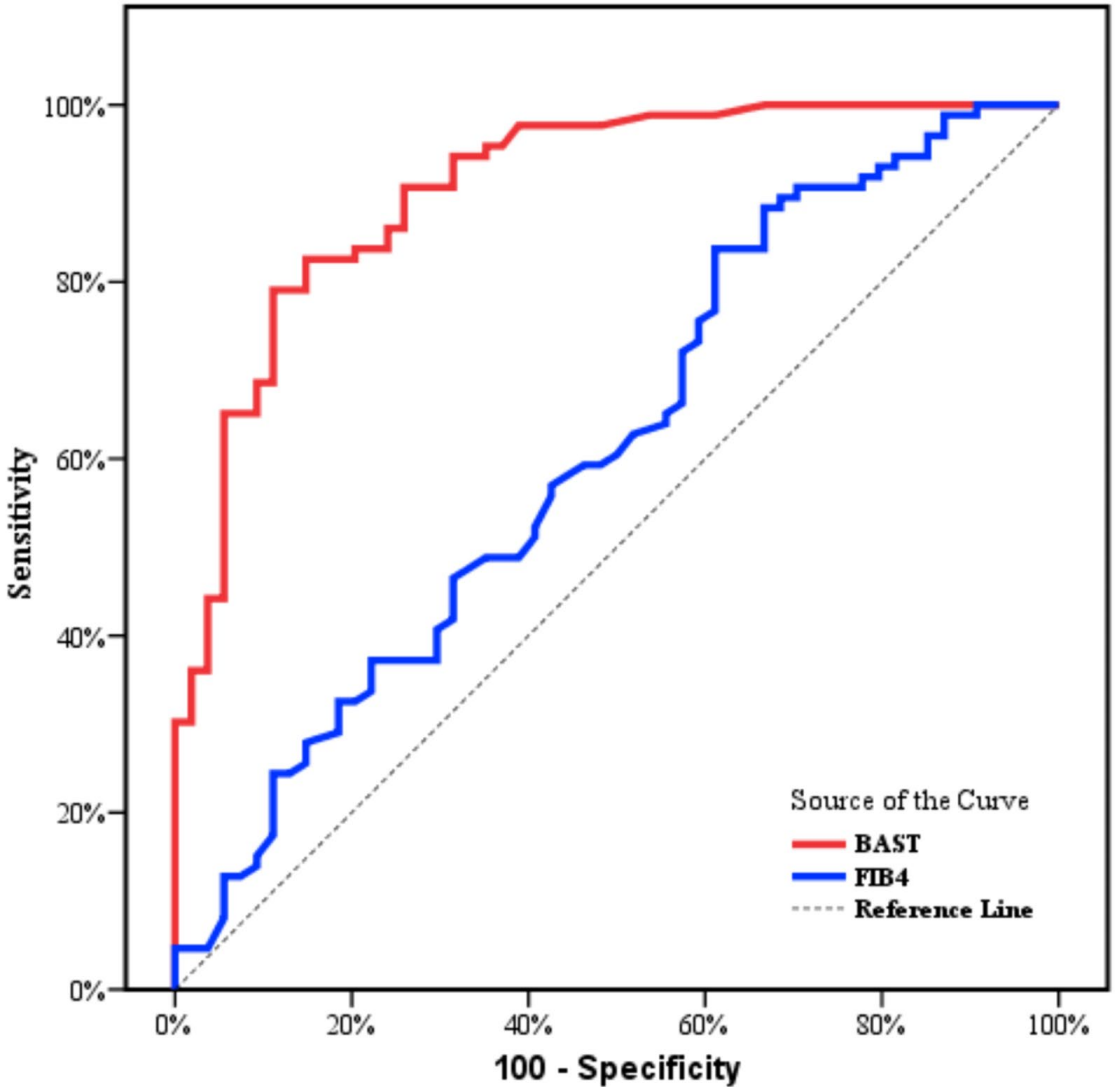
ROC curve for BAST and FIB4 to discriminate F1–F2 from F3–F4

Table 3 and Fig. 1 show the diagnostic performance of BAST and FIB-4 to discriminate (F1–F2) from (F3–F4). At cutoff point ≤ − 0.451, the BAST score had a sensitivity of 90.70% and specificity of 74.07% with PPV 84.80%, NPV 83.30%, and AUC of 0.907 showing that all indices were superior to that of FIB-4 where was at cutoff point ≤ 1.24, FIB-4 index had a sensitivity 60.47% and specificity 50.00% with PPV of 65.80%, NPV of 44.30% and AUC 0.614.

**Table 3:** Diagnostic performance of BAST score and FIB-4 index to discriminate (F1–F2) from (F3–F4)

	AURC	95% CI	Cut off	Sensitivity	Specificity	PPV	NPV	*p*
BAST	0.907	0.857–0.957	≤ − 0.45	90.70	74.07	84.80	83.30	< 0.001
FIB4	0.614	0.517–0.711	≤ 1.24	60.47	50.00	65.80	44.30	0.024

*AURC* areas under the receiver operating characteristic curves; *CI* confidence intervals, *NPV* negative predictive value, *PPV* positive predictive value

**Fig. 2:**
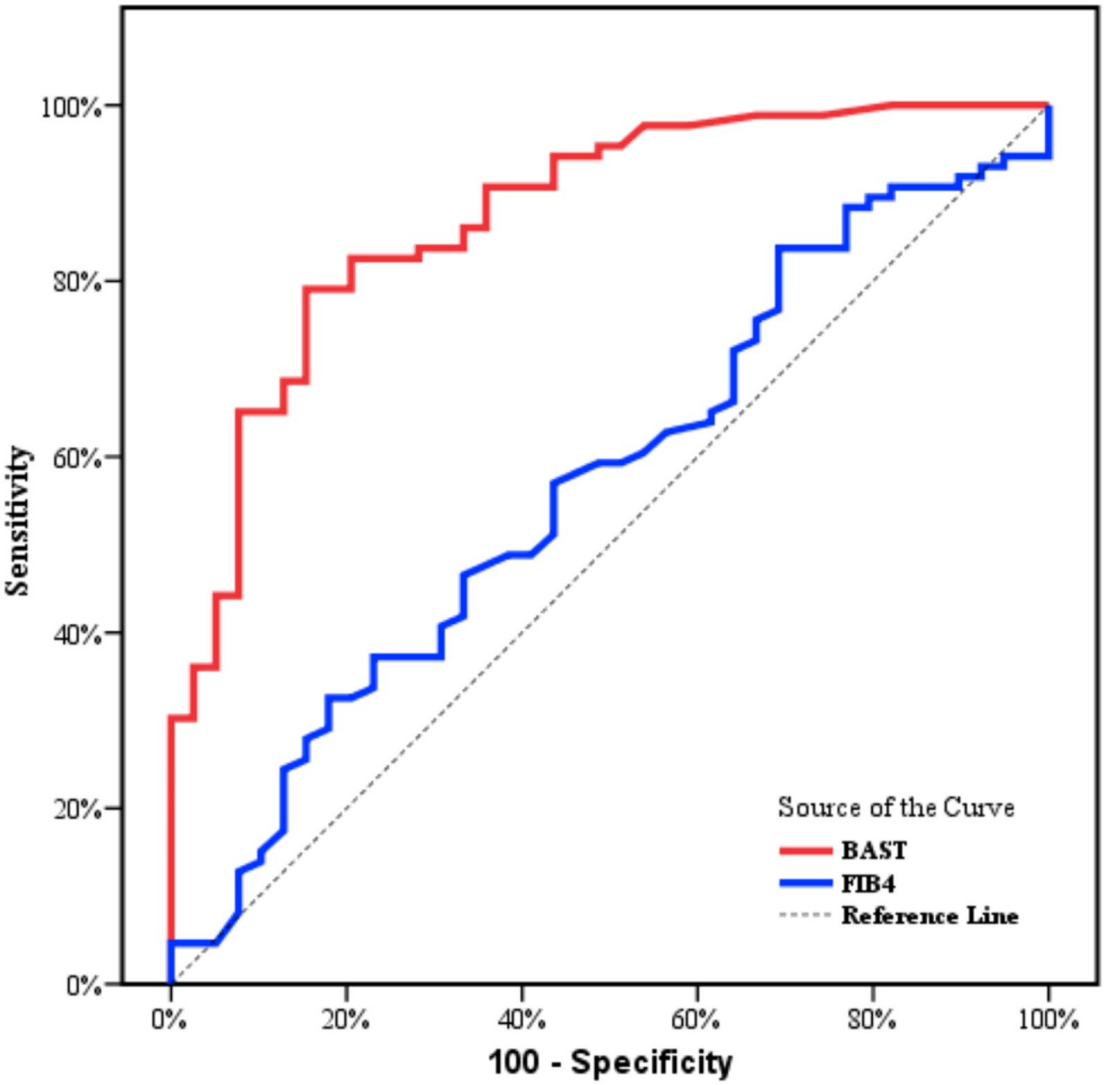
ROC curve of BAST and FIB-4 to discriminate F1 from F2

Table 4 and Fig. 2 show the diagnostic performance of BAST and FIB-4 to discriminate (F1) from (F2). At cut off point ≤ − 1.11, the BAST score had a sensitivity of 80.23%, specificity of 79.49, PPV 89.60%, NPV 64.60%, and AUROC was 0.873, while, at the cutoff point ≤ 1.21, FIB-4 had a sensitivity of 59.30% and specificity 51.28%, PPV 72.90%, NPV 36.40% and AUR was 0.570.

**Table 4:** Diagnostic performance of BAST and FIB-4 to discriminate F1from F2

	AURC	95% CI	Cut off	Sensitivity	Specificity	PPV	NPV	*p*
BAST	0.873	0.807–0.939	≤ − 1.11	80.23	79.49	89.60	64.60	< 0.001
FIB4	0.570	0.463–0.678	≤ 1.21	59.30	51.28	72.90	36.40	0.209

*AURC* areas under the receiver operating characteristic curves, *CI* confidence intervals, *NPV* negative predictive value, *PPV* positive predictive value

## Discussion

Currently, the most common cause of chronic liver disease, hepatocellular carcinoma, and liver transplantation is MASLD [3]. In MASLD, the extent of hepatic fibrosis is a critical risk factor for predicting clinically meaningful outcomes. For MASLD patients, algorithms that evaluate high-risk groups for severe hepatic fibrosis are therefore essential. The “gold standard” for diagnosing and staging hepatic fibrosis involves combining a liver biopsy with a histological assessment [17]. However, liver biopsy is an intrusive technique that comes with a number of disadvantages [5]. Numerous studies investigating non-invasive techniques for assessing hepatic fibrosis, such as imaging and serologic testing, have been conducted in the past few years [18].

Liver stiffness measurement using transient Elastography is a highly accurate noninvasive marker for advanced fibrosis and has prognostic value [19, 20]. While LSM is a cost-effective screening tool, its availability in primary care settings is limited [21].

Furthermore, FibroScan overestimates the fibrosis score in the early stages of fatty liver, whereas it has high accuracy in detecting advanced fibrosis and cirrhosis. Hence, it can be concluded that transient Elastography is a good adjunctive tool in fatty liver patients with advanced fibrosis [22]. FIB-4 has been suggested as an affordable alternative for initial screening [21]. However, a study by Graupera et al. found that FIB-4 may not be optimal for screening due to a risk of overdiagnosis and false negatives, particularly in patients with chronic liver disease risk factors. In addition, the authors suggested waist circumference as a potential initial step in identifying individuals at risk for liver fibrosis in the general population [23].

A recently created score called the BAST score is used to evaluate the level of liver fibrosis in patients with diabetic NAFLD. In diabetic patients, the score was found to predict NAFLD fibrosis more accurately than FIB-4 and NAFLD fibrosis scores. The score was designed and validated internally to predict the existence of liver fibrosis. Waist circumference, BMI, and AST level all make the BAST score [9].

There are several logical reasons for the rationale behind the BAST score’s assessment of fibrosis severity in MASLD patients. First, the score is influenced by BMI. As expected, there is a considerable correlation between incident MASLD/steatohepatitis and BMI [22].

In addition, prior research has linked obesity to an increased risk of incident cirrhosis [24]. In addition, a recent big study involving over 2.1 million participants discovered a robust and remarkable near-linear association between BMI and the likelihood of being diagnosed with steatohepatitis [25]. Second, waist circumference was one of the elements in the BAST score. Well-known risk factors for MASLD [26, 27] include waist circumference and body mass index, which are often used indicators for detecting obesity or abdominal obesity. In addition, the majority of research indicated that having a high-fat mass could help predict the incidence of MASLD [28, 29]. The third is that the score includes the liver enzyme (AST). Liver enzymes are recognized to be markers of hepatocellular injury, and numerous studies have shown a link between elevated liver enzyme levels and an increased risk of steatohepatitis. It was discovered that the AST either stays constant or increases while the ALT usually decreases in fatty liver patients with growing hepatic fibrosis [30]. Furthermore, a number of clinical scoring schemes based on straightforward clinical or laboratory markers have been put forth to help fatty liver patients identify severe fibrosis. AST/ALT ratio and the aspartate aminotransferase (AST)-to-platelet ratio index (APRI) are two examples of these [31].

The BAST score and FIB-4 were compared in this study as a non-invasive way to identify the degree of liver fibrosis in MASLD patients. However, we acknowledge that the NAFLD Fibrosis Score (NFS) is another well-established non-invasive tool for assessing liver fibrosis. The decision to focus on the FIB-4 index was based on its simplicity and widespread use in clinical practice. Many researchers have looked at the diagnostic performance of the FIB-4 in patients with fatty liver disease (FLD) [32, 33]. Currently, most practice guidelines recommend using the NFS and FIB-4 as the initial step in identifying high-risk groups among obese patients with FLD. The specificity of FIB-4 for severe fibrosis decreases with age, reaching less than 30% in people over 65 years. Other limitations of FIB-4 include its limited specificity and positive predictive value for hepatic fibrosis [33]. This may cause these elderly patients to receive false-positive results [35]. Additional factors that change the AST, ALT, or platelet count could lead to an incorrect FIB-4 score because the score is based on laboratory results. For example, drugs that increase AST, such as alcohol, can also increase the FIB-4 score, potentially producing false positives. False positives could occur if people with low platelets from non-portal hypertension-related causes also overestimate FIB-4 [36].

In this study, we conducted ROC analyses to assess the diagnostic performance of the BAST score and FIB-4 in distinguishing between advanced cirrhosis (F3–F4) and mild–moderate fibrosis (F1–F2). The results showed that the BAST score had a sensitivity of 90.70% and specificity of 74.07% at a cutoff of ≤ − 0.451, while FIB-4 had a sensitivity of 60.47% and specificity of 50.0% at a cutoff of ≤ 1.24. This indicates that the BAST score is more accurate in identifying advanced fibrosis/cirrhosis compared to FIB-4, which was previously identified as a weakness in FIB-4 [8, 23]. In addition, this study found that the BAST score outperformed the FIB-4 score in diagnosing advanced fibrosis in NAFLD patients, where the BAST score had a higher PPV of 84.8%, NPV of 83.3%, and AURC of 0.907 compared to the FIB-4 score with PPV of 65.8%, NPV of 44.3%, and AURC of 0.614. So, the BAST score shows promise for accurate diagnosis of advanced fibrosis in high-risk Groups, which is a weak point in FIB-4 [34, 35].

In addition, to distinguish F1 (mild fibrosis) from F2 (moderate fibrosis) in this study, BAST score had sensitivity of 80.23%, specificity of 79.49, PPV89.6%, NPV 64.6%, and AUROC was 0.873 while, at cutoff point ≤ 1.21, FIB-4 had sensitivity of 59.30% and specificity of 51.28%, PPV 72.9%, NPV % 36.4, and AUR of 0.570. Once more, these data show that, in comparison to the FIB-4, the BAST score has adequate accuracy to be used to diagnose mild from moderate liver fibrosis. Our research, in line with a multicentre study, found that FIB-4 has limited accuracy in detecting liver fibrosis due to a high risk of overdiagnosis and a significant number of false negative results [32, 36].

This study has limitations that warrant consideration. First, the relatively small sample size comprised exclusively of Egyptian patients limits the generalizability of findings to broader populations with diverse genetic backgrounds and lifestyles. Thus, a validation study involving patients from diverse racial and ethnic backgrounds is required. Exclusion of individuals with DM based on past medical history alone, without measuring HbA1c, may have omitted undiagnosed diabetic cases, potentially affecting the applicability of results to all MASLD patients. The inclusion of waist circumference (WC) as a variable in the BAST score requires further discussion. While there is evidence linking abdominal obesity to the risk of MASLD and liver fibrosis. Waist circumference is a reliable marker for abdominal obesity, closely tied to metabolic dysfunction and hepatic steatosis. Studies consistently show that higher waist circumference is associated with increased risk of liver fibrosis and metabolic issues [24, 25, 28, 29]. However, WC prevents the score from being used in automated pathways, and it cannot be reported in a laboratory report in the same ways as FIB-4 so it, Hindersing the score’s use in automated pathways. Furthermore, various protocols exist for measuring waist circumference, leading to variable accuracy based on who measures it [37]. Despite these challenges, including waist circumference in the BAST score offers valuable insights into abdominal obesity and liver fibrosis risk.

This study highlights the importance BAST score in accurately screening for MASLD fibrosis, a serious condition that can lead to liver complications and increased mortality rates, to identify individuals who may benefit from new drug therapies such as resmetirom. The recent FDA approval of resmetirom offers hope for treating non-cirrhotic MASLD with promising results in clinical trials. Resmetirom targets the liver and has shown effectiveness in reducing hepatic fat, improving liver health, and reducing biomarkers of liver damage without affecting weight or glucose metabolism [38].

Future research should focus on developing standardized measurement protocols and integrating waist circumference data into automated clinical pathways.

## Conclusions

This study underscores the diagnostic utility of the BAST score compared to the FIB-4 index in predicting liver fibrosis severity among non-diabetic patients with MASLD. The BAST score demonstrated superior performance in distinguishing advanced fibrosis/cirrhosis from mild to moderate fibrosis, as evidenced by its higher sensitivity, specificity, PPV, NPV, and AUC. However, future studies with larger, more diverse cohorts and comprehensive diagnostic protocols are necessary to validate the robustness and generalizability of the BAST score in clinical practice.

## Data Availability

The data used in this study is available upon a reasonable request from the corresponding author and after permission from all participating services.

## Abbreviations

NAFLD: Non-alcoholic fatty liver disease
MAFLD: Metabolic dysfunction-associated fatty liver disease
MASLD: Metabolic dysfunction-associated liver disease
NASH: Non-alcoholic steatohepatitis
FLD: Fatty liver disease
CBC: Complete blood count
FIB4: Fibrosis index based on 4 factors
NFS: NAFLD fibrosis score
CAP: Controlled attenuation parameter
LSM: Liver stiffness measurement
BMI: Body mass index
